# Introducing Machine Perfusion into Routine Clinical Practice for Liver Transplantation in the United States: The Moment Has Finally Come

**DOI:** 10.3390/jcm12030909

**Published:** 2023-01-23

**Authors:** Kristopher P. Croome

**Affiliations:** Department of Transplant, Mayo Clinic Florida, Jacksonville, FL 32224, USA; croome.kristopher@mayo.edu; Tel.: +1-904-956-3261

**Keywords:** perfusion, pumping, DCD, HMP, NMP, D-HOPE, NRP, TA-NRP

## Abstract

While adoption of machine perfusion technologies into clinical practice in the United States has been much slower than in Europe, recent changes in the transplant landscape as well as device availability following FDA approval have paved the way for rapid growth. Machine perfusion may provide one mechanism to maximize the utilization of potential donor liver grafts. Indeed, multiple studies have shown increased organ utilization with the implementation of technologies such as ex-situ normothermic machine perfusion (NMP), ex-situ hypothermic machine perfusion (HMP) and in-situ normothermic regional perfusion (NRP). The current review describes the history and development of machine perfusion utilization in the Unites States along with future directions. It also describes the differences in landscape between Europe and the United States and how this has shaped clinical application of these technologies.

In the United States (USA), there continues to be a discrepancy between the number of patients awaiting liver transplantation and the number of available donor livers [1]. As the transplant community continues to look for novel solutions to address this problem, new technologies such as machine perfusion may provide one mechanism to maximize the utilization of potential donor liver grafts. Indeed, multiple studies have shown increased organ utilization with the implementation of technologies such as ex situ normothermic machine perfusion (NMP), ex situ hypothermic machine perfusion (HMP) and in situ normothermic regional perfusion (NRP) [2,3,4]. While these technologies have been widely adopted in Europe, their adoption in the United States has been slower for a myriad of reasons. The present review describes the development and implementation of machine perfusion technologies into clinical practice in the United States.

## 1. Potential Benefits of Machine Perfusion Technologies

Since the initial rise of liver transplantation, static cold storage (SCS) has remained the gold standard in preserving donor livers due to its low cost and simplicity. While this form of preservation has worked well for high-quality organs with relative short cold ischemic times, it has several limitations particularly when trying to expand the donor pool with donation after circulatory death (DCD) or other “marginal” livers. These limitations include: (I) sustained organ injury not being reversed; (II) further organ injury during storage; (III) organ viability cannot be assessed; and (IV) storage time is limited [5]. Machine perfusion technologies have the potential to ameliorate organ ischemic injury at the time of procurement, allow for viability testing, increase storage time and minimize further injury during storage.

Initial studies from Europe have demonstrated encouraging results for NMP, HMP and NRP. The safety of NMP was initially demonstrated by the Oxford transplant group using the OrganOx metra device [6], after which Nasralla et al. published the first randomized controlled trial comparing the efficacy of NMP vs. SCS in liver transplantation [7]. Compared to SCS, the NMP group demonstrated a 49.4% reduction in the peak level of serum aspartate transaminase (AST) during the first 7 days after liver transplantation (*p* < 0.0001). The effect was most pronounced in DCD livers, where the peak AST was reduced by 73.3% compared to 40.2% in donation after brain death (DBD) livers. In addition, there was a 50% lower organ discard rate and 54% longer mean preservation time in the NMP group.

When looking at ex situ HMP, a multicenter study from transplant centers in Zurich and Birmingham compared DCD liver grafts treated with SCS, with livers treated with 1–2 h of hypothermic oxygenated perfusion (HOPE) after cold storage. In that study, graft loss was significantly less in HOPE-treated livers, despite longer donor warm ischemia times than SCS. [8]. More recently, a multicenter RCT trial investigating HMP in DCD liver transplantation was performed (DHOPE-DCD Trial) [9]. In that trial, 78 patients received a HMP liver and 78 received a liver following SCS alone. Early allograft dysfunction (EAD) occurred in 26% of the machine-perfused livers, compared with 40% of the SCS livers (risk ratio, 0.61). Non-anastomotic biliary strictures occurred in 6% of the patients in the machine-perfusion group and in 18% of those in the SCS group (risk ratio, 0.36; *p* = 0.03).

In situ NRP represents a different technology to ex situ NMP and HMP. NRP provides oxygenated perfusion and the potential recovery of the organs in DCD donors where a mandatory period of warm ischemia has occurred during the withdrawal of life-sustaining therapy (WLST). Previous studies looking at in situ NRP have demonstrated reduced ischemic cholangiopathy (IC) and improved patient and graft survival compared to SCS [10]. In addition, increased liver utilization has been demonstrated compared to SCS [4].

While high-quality data from randomized trials demonstrating a clear benefit of machine perfusion technologies compared to SCS is still limited to a few trials, there is growing literature from non-randomized studies to suggest potential benefits, particularly for DCD and other “marginal” donor livers.

## 2. Why Machine Perfusion Has Been Slower to Be Adopted in USA Than Europe?

The slower adoption of machine perfusion in the USA than Europe is multifactorial; however, the primary reason has been the lacking clinical availability until after U.S. Food and Drug Administration (FDA) approval. The first FDA approval for an ex situ perfusion device was on 28 September 2021 [11]. Prior to that date, ex situ machine perfusion was only available in the USA on protocol in one of the registered randomized control trials. Currently there are only two ex situ machine perfusion devices that are FDA approved for clinical use.

Ex situ machine perfusion devices have been approved for clinical use in Europe for some time. Many of the first machine perfusion platforms were developed in Europe. In addition, the medical device approval process has previously been shown to occur much more quickly in Europe than in the United States [12]. The FDA approval process is generally lengthier, as clinical trials are usually required to demonstrate that products are reasonably safe and effective. In the European Union, by contrast, medical devices can be sold if they perform “as intended” and “are likely to be safe”. These differences in process have generally resulted in medical devices getting to market faster in Europe than in the USA.

Another challenge to using machine perfusion in the USA has been the large land area over which organ procurement take place. The land area of the USA is 3.80 million miles^2^ compared to 0.09 million miles^2^ in the United Kingdom. As a result of this vast area, many organ procurements require plane flights, a situation that is even more pronounced when looking at marginal liver grafts [13]. This requires transport of a capable team and equipment to the location of the donor hospital (except in the case of back-to-base). Any medical device must be approved by the Federal Aviation Administration (FAA) in addition to the FDA, if it is to be transported in the air. While the large land area of the USA may present an initial barrier to the adoption of machine perfusion, it may also lend itself to the advantages of machine perfusion, where longer organ preservation times are possible [14,15,16,17].

## 3. Current Pressures on Organ Donation and Why They Have Increased the Push for Machine Perfusion in the USA

Organ Procurement Organizations (OPOs) are not-for-profit organizations responsible for recovering organs from deceased donors for transplantation in the USA. There are 57 OPOs, each mandated by federal law to perform this mission in the donation service area (DSA) assigned to them. As part of an executive order, *Advancing American Kidney Health*, by President Donald Trump, changes to the organ procurement system were sought to help the almost 109,000 people in the USA currently on the transplant list for a lifesaving organ [18]. On November 20, 2020, the Centers for Medicare & Medicaid Services (CMS) issued a final rule that updated the donation rates and transplant rates for OPOs that must be met to receive Medicare and Medicaid payments [19]. The goal of this final rule was to capture the full potential of donor organs and to hold poorly performing OPOs to national standards. These new conditions went into effect on 1 August 2022 and placed greater pressure on OPOs to ensure that donor organs are maximally utilized for organ donation. The results of this mandate remain to be determined; however, the push to minimize organ non-utilization lends itself to technologies such as machine perfusion, which may increase utilization, particularly for marginal livers [2,3,4].

In addition to the pressures on OPOs to increase organ utilization, there have also been significant changes to the liver allocation process that have increased the pursuit of “marginal” liver grafts by transplant centers. Following years of debate and legal challenges from all sides, a liver distribution system based on acuity circles went into effect on 4 February 2020 [20]. This acuity circle allocation system replaced DSA and regional boundaries previously used in liver organ distribution with a system based on distance between donor hospital and transplant hospital. This system resulted in broader sharing of livers and substantially increased both the distances traveled and the costs for organ retrieval. Since implementation of this policy change, waiting times have decreased for patients with a MELD score of ≥29, while waiting times have increased for those patients with a MELD score of ≤28 [21]. This has placed pressure on transplant programs to increasingly pursue DCD and other “marginal” livers for patients listed with a MELD score of ≤28 [21]. In addition to the recent implementation of the acuity circles system, HCC patients no longer have a “ladder” model of increasing exception scores over time and instead are given an exception score of median MELD at transplant minus 3 (MMaT-3) for a 250 mile radius surrounding the donor location. This has significantly reduced access to standard criteria livers for patients with HCC. As a result, the utilization of DCD livers for patients with HCC has significantly increased [22]. Given the increased pursuit of marginal liver grafts, many transplant programs have shown interest in using various machine perfusion technologies to help ameliorate the risks associated with these livers [23].

## 4. Machine Perfusion Platforms Available Commercially or as Part of a Clinical Trial in the USA

**Figure 1:**
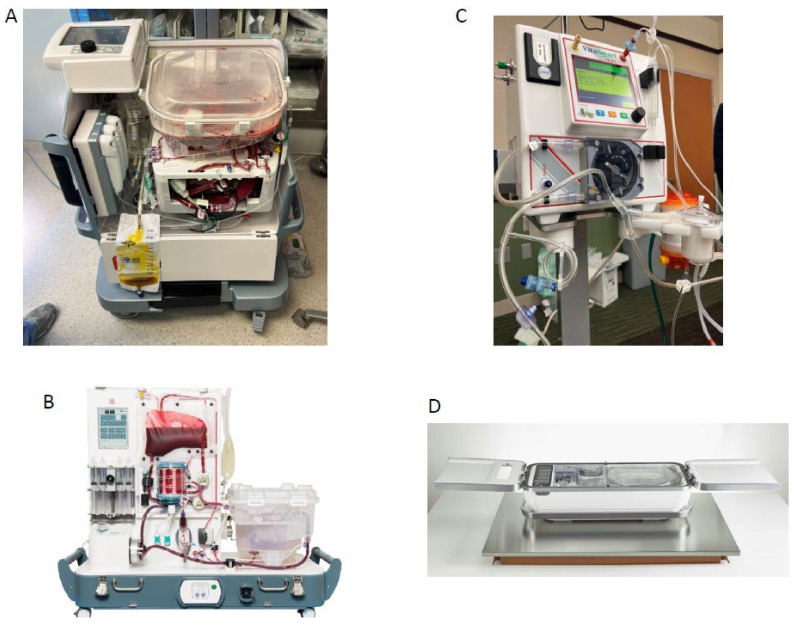
Machine Perfusion Platforms Available Commercially or as part of a Clinical Trial in the USA. (**A**) OCS Liver System (TransMedics); (**B**) OrganOx Metra System; (**C**) VitaSmart Liver Machine Perfusion System (Bridge to Life); (**D**): LifePort Liver Transporter (LLT) System.

The following are the NMP and HMP platforms that are either FDA approved for clinical use or currently in the clinical trial stage in the USA (Figure 1):

- OCS liver system (TransMedics): NMP, FDA approved.
- OrganOx metra^®^ System: NMP, FDA approved.
- LifePort Liver Transporter (LLT) system: HMP, DHOPE, clinical trial complete with FDA approval pending.
- VitaSmart Liver Machine Perfusion System (Bridge to Life): HMP, HOPE via portal vein alone, clinical trial currently recruiting.

## 5. Ex situ Normothermic Machine Perfusion in the USA

Prior to September 2021, the utilization of NMP was only available for clinical use as part of a registered clinical trial. The Liver PROTECT trial compared livers treated on the OCS liver system (TransMedics) compared with SCS controls [24]. This study was conducted between November 2016 and October 2019 at 20 US liver transplant programs. Final results demonstrated a significant reduction in EAD with OCS (OCS 18% vs. SCS 31%, *p* = 0.009). OCS use was also associated with a significant reduction in ischemic biliary complications at one year (OCS 2.6% vs. SCS 9.9%, *p* = 0.019). Patient survival at one year was high, at 94% for both OCS and SCS control arms [25].

A multicentered randomized control trial comparing the OrganOx NMP system with SCS was conducted between 9 October 2016 and 3 February 2020 at 15 US liver transplant programs [26]. While data from that study has not been published in a peer reviewed journal, the study data is available through the FDA approval document [27]. Results demonstrated no significant difference in EAD between the OrganOx (18.7%) and SCS (24.9%) groups. Twelve-month graft survival rates were 97.0% and 97.7% in the NMP and SCS arms, respectively [27].

Several single-centered trials investigating NMP have been performed at Cleveland Clinic using their internally developed NMP platform [28]. Data from one of those trials looking at discarded livers demonstrated that 16 (71.5%) of the discarded livers placed on NMP were successfully transplanted after organ perfusion and assessment using an institutionally built device. Seven livers had EAD, and one patient developed IC after 4 months and was treated with biliary stents. All other patients had good liver function, with a follow-up time of 8 weeks to 14 months [29].

The OCS liver system (TransMedics) received FDA approval for clinical use on 28 September 2021, while the OrganOx metra^®^ System received FDA approval for clinical use on 9 December 2021. Since those dates, the clinical use of both platforms has increased. The OCS liver system (TransMedics) may be used directly by members of the transplant center or may be used as an “end-to-end” service through the National OCS Program run by TransMedics. The National OCS Program employs procurement surgeons that will procure and place the donor liver on NMP, then fully staff the device during transport until implantation of the liver at the recipient transplant center [3]. The transplant center will then be charged a set fee for the device and these services. The OCS liver system is not supported for back-to-base usage. The OrganOx metra^®^ System is available for use by members of the transplant center. OrganOx does not offer a national program. The OrganOx metra^®^ System is the only NMP device in the US that supports back-to-base usage, where the liver is procured and brought back to the transplant center using SCS and then placed on NMP.

At the time of writing this manuscript, there have been no publications on the outcomes of NMP in the USA following FDA approval of the two aforementioned devices. There have been several publications using data from the Scientific Registry of Transplant Recipients (SRTR) that looked at NMP outcomes from liver transplants that were performed as part of clinical trials. The first study investigated N = 228 livers that underwent NMP (OCS or OrganOx) prior to liver transplant. The NMP group had a 3.5% discard rate versus 13.3% in the SCS group (*p* < 0.001) [4]. Survival analysis, following propensity score matching, found no significant difference in 1-year overall survival between recipients of NMP versus COLD livers. The second study investigated a total of 374 cases in which liver grafts underwent ex situ machine perfusion prior to LT (OCS, n = 218; OrganOx, n = 110; LifePort, n = 23; and other, n = 23). Graft survival at 1, 2, and 3 years was 90.1%, 89.1%, and 87.0% in patients who underwent LT following ex situ machine perfusion. Graft failure within 30 days occurred in eight (2.1%) patients [30].

## 6. Ex situ Hypothermic Machine Perfusion in the USA

Currently there are no HMP devices that are FDA approved for clinical use in the USA. HMP in the United States was initially carried out by Guarrera et al. at Columbia University [31]. In this first clinical series, 20 livers that had undergone HMP and 20 SCS livers utilized for liver transplant between July 2004 and February 2008 were compared. There were no cases of primary nonfunction in either group. EAD rates were 5% in the HMP group versus 25% in controls (*p* = 0.08). In a follow-up to that study, the same group published data from the registered trial: Hypothermic Machine Preservation-Phase 2 [32]. In that study, there were significantly fewer biliary complications in the HMP group versus the SCS group (4 vs. 13, *p* = 0.016). Mean hospital stay was significantly shorter in the HMP group (13.64 ± 10.9 vs. 20.14 ± 11.12 days in the SCS group, *p* = 0.001). [33].

A multicentered randomized control trial comparing the LifePort Liver Transporter (LLT) system using Vasosol^®^ with SCS was conducted between 3 April 2019 and 30 July 2022 at nine US liver transplant programs [34]. Results from this study have yet to be published. Another multicentered randomized control trial comparing the VitaSmart Liver Machine Perfusion System (Bridge to Life) with SCS commenced recruitment on 16 December 2021 at 15 US liver transplant programs and is still actively recruiting [35].

## 7. Normothermic Regional Perfusion in the USA

When discussing NRP it is important to differentiate between Abdominal NRP (A-NRP) and Thoracoabdominal-NRP(TA-NRP). While these procurement techniques share the utilization of extracorporeal membrane oxygenation, they differ dramatically in their cannulation techniques, which organs are provided with in situ perfusion, and the re-initiation of cardiac activity. A-NRP provides in situ perfusion of the abdominal organs only via the infrarenal aorta and IVC (or alternatively the femoral artery and vein) and does not involve the reinitiation of cardiac activity, while TA-NRP involves in situ perfusion of the thoracic and abdominal organs (via the aortic arch and right atrium) as well as the reinitiation of cardiac activity.

The first group to publish their experience with A-NRP in the US was the University of Michigan. In that experience, the authors described 37 cases of A-NRP in which livers were used for transplantation in 13 cases [36]. The Michigan A-NRP program was active from 2000–2013. One- and two-year graft-survival rates were 85.7% and 71.4%, respectively. IC was reported in one patient (14.3%).

The use of NRP in the US was largely abandoned until 2020. As DCD heart transplantation started to gain support, select thoracic transplant programs began to pursue TA-NRP as an alternative to ex situ machine perfusion in establishing a viable means to procure hearts from DCD donors. An initial series of 15 cases of TA-NRP was published by a group from Vanderbilt and clinical trial protocols were listed from both NYU and University of Nebraska [37]. These TA-NRP cases were driven by the thoracic teams procuring the DCD hearts; however, in these cases, livers were also procured by abdominal teams and utilized for liver transplantation. Several publications have looked at the outcomes of these DCD livers procured through TA-NRP. The first study investigated the outcomes of 24 LTs in which TA-NRP was used [30]. Median post-LT follow-up was 4.75 months. Out of the 24 cases, there was one LT recipient death at 6.2 months with cause of death listed as heart failure and shock. None of the patients undergoing LT with a liver procured through TA-NRP were re-listed at the time of maximal follow-up. A second study looked at 13 cases of LT in which TA-NRP was used [38]. In that study, the rate of EAD was 23% and three of the transplant recipients received a simultaneous liver-kidney (SLK). In the three SLK cases, none of the recipients had EAD or DGF. One recipient died 186 days post-transplant from sepsis but had normal pre-sepsis liver function. No recipients developed clinical evidence of IC and 12 of 13 (92%) patients were alive with good liver function at 439 days median follow-up.

There is renewed interest in the establishment of A-NRP in the USA driven by abdominal transplant teams. At Mayo Clinic Florida, we began the development of an A-NRP program in January 2022. To date, we have performed a total of 10 liver transplants using A-NRP recovery in the donor. Six-month graft and patient survival are 100% and we have had no cases of IC. The rate of EAD has been 10% and the PRS has been 0%, compared to a rate of 33.6% and 26.0%, respectively, in our DCD LT undergoing SCS [39].

Discussions surrounding the ethics of NRP in the United States has resulted in several individuals and groups wanting further conversation on the topic. These discussions have largely been in reference to TA-NRP where the heart is restarted, and the brachiocephalic vessels are clamped to prevent reperfusion of the brain. They have not been raised regarding A-NRP, where only the abdominal organs receive in situ perfusion. The most notable of these groups wanting further conversation on TA-NRP was a white paper by the American College of Physicians [40]. Subsequently, several editorials were published taking the position that TA-NRP is consistent with US ethical and legal standards [41,42].

The American Society of Transplant Surgeons (ASTS) released a statement addressing TA-NRP in DCD donors on 23 August 2022 [43]. In that statement, the ASTS supported the ethical acceptability of TA-NRP as meeting the ethical baseline for DCD organ donation. Their analysis on the specific ethical concerns of TA-NRP were as follows:

- **Perfusion of the organs in the body**: This is not autoresuscitation or resuscitation of the donor. The donor is dead before the initiation of NRP. NRP is mechanically assisted regional perfusion and oxygenation of organs for transplantation.
- **Clamping the brachiocephalic vessels** before the initiation of TA-NRP ensures that the brain is not reperfused. Circulatory death has already been determined under the UDDA and in accordance with accepted medical standards when the brachiocephalic vessels are clamped.
- **Reperfusing the heart** in the body on NRP is no different that restarting the heart outside of the body with machine perfusion. While this is optically different, in both cases, the heart is restarted with artificial machine assistance for the purpose of organ donation in a person who died intending to donate their organs. The heart would not continue to function within the donor without ventilatory support, so it is functioning only with mechanical assistance for the purposes of organ donation.

In addition to the above statement, the ASTS also released recommendations on best practices in DCD organ procurement, that was supportive of both TA-NRP and A-NRP [44].

## 8. The Future of Machine Perfusion in the USA

The interest in machine perfusion in the USA is strong, particularly as the utilization of DCD donors has continued to rise [45]. As in Europe, questions remain on which technologies (HMP, NMP or NRP) will gain the widest adoption in clinical practice. Costs of these new technologies vary drastically. There is no question that in addition to effectiveness, cost will also likely have a significant influence on more widespread adoption. Like all new technologies in healthcare, the question of how the costs of these technologies will be addressed remains uncertain. In the USA, healthcare coverage is generally either through private payor insurance or through Medicare and Medicaid services. Medicare is a medical insurance program for people over 65 and younger disabled people and dialysis patients. Medicaid is an assistance program for low-income patients’ medical expenses. Private payor reimbursement is generally a contracted global rate for liver transplantation with certain “outlier” reimbursements for cases that far exceed the global rate. These global rates are generally contracted with private payers at set intervals; for example, every 1 or 2 years, etc. Whether private payers will be willing to pay the increased costs associated with organ acquisition using machine perfusion when contracts come up for renegotiation, remains to be determined. It is likely the transplant community will need to justify the benefit of using these devices both in terms of cost and benefit to patients.

For Medicare and Medicaid patients, reimbursement for organ acquisition costs (which would include the costs of machine perfusion) would fall under the *Cost Report* for all Medicare patients. This reimbursement is based on the percentage of Medicare and Medicaid patients receiving liver transplantation at the respective transplant program and that percentage of the total *Cost Report* is reimbursed. Previous authors have speculated that for Medicare and Medicaid patients, the costs of machine perfusion will be fully reimbursed under the organ acquisition *Cost Report* for all Medicare patients [3]. Many uncertainties remain in determining exactly how reimbursement for machine perfusion will function. The US healthcare system is very complex and a true explanation of how the system functions financially is well beyond the scope of this paper. In the future, it will be necessary to fully define the reimbursement process in order for machine perfusion to gain widespread usage throughout the USA.

While still in the experimental phase, it is possible that in the future machine perfusion protocols could employ gene therapy, nanoparticles, or liver defatting. These protocols could potentially allow for the more extensive reconditioning of damaged livers [46,47].

In conclusion, while adoption of machine perfusion technologies into clinical practice in the United States has been much slower than in Europe, recent changes in the transplant landscape as well as device availability following FDA approval have paved the way for rapid growth. The future of these technologies in the USA is exciting, as many are hopeful they will facilitate the maximal utilization of donor livers, thus honoring the wishes of the donor and the donor’s family to provide a lifesaving gift.

## Data Availability

Not applicable.

## Abbreviations

CI	Confidence interval
CIT	Cold ischemia time
CT	Computed tomography
DCD	Donation after Circulatory Death
DBD	Donation after Brain Death
D-HOPE	Dual dual portal vein and hepatic artery hypothermic oxygenated perfusion
DRI	Donor risk index
HCC	Hepatocellular carcinoma
HMP	Hypothermic Machine Perfusion
KDPI	Kidney donor profile index
LT	Liver transplant
MELD	Model for end stage liver disease
NMP	Normothermic Machine Perfusion
NRP	Normothermic Regional Perfusion
OCS	Organ Care System
OPO	Organ procurement Organization
OPTN	Organ Procurement Transplant Network
RCT	Randomized Control Trial
SD	Standard deviation
STAR	Standard Analysis and Research
TA-NRP	Thoraco-abdominal Machine Perfusion
UNOS	United Network for Organ Sharing
